# An Innovative Miniature Bite Force Recorder

**DOI:** 10.5005/jp-journals-10005-1093

**Published:** 2010-04-15

**Authors:** Sarabjeet Singh, Ashok K Utreja, Navreet Sandhu, Yadvinder S Dhaliwal

**Affiliations:** 1 Professor and Head, Department of Orthodontics, National Dental College, Dera Bassi, Punjab, India; 2 Professor and Head, Unit of Orthodontics, Division of Oral Health Sciences, PGIMER, Chandigarh, India; 3 Reader, Department of Prosthodontics, National Dental College, Dera Bassi, Punjab, India; 4 Assistant Professor, Department of Orthodontics, National Dental College, Dera Bassi, Punjab, India

**Keywords:** Gnathodynamometer, Bite force, Myoelectric activity, Laboratory calibration test.

## Abstract

In this study, a detailed description of development of a new novel bite force recorder (gnathodynamometer) using solid state components is vividly explained. This state of the art authenticated device can be used to assess the complex function of human bite force, which is the net resultant combination of functional response of various craniomandibular structures consisting of interrelated components, like the muscles of mastication, joints, teeth and the neuromuscular system. The consistency and accuracy of the bite force recorder was reaffirmed by doing a detailed laboratory calibration and clinical testing on 30 adult subjects.

## INTRODUCTION

The craniomandibular function is determined by the complex and interrelated components comprising the morphology and biomechanics of the muscles, joints, teeth and the neuromuscular system.^1^

Bite forces, which greatly differ in magnitude and direction, result from different combinations of action of masticatory and cooperative muscle.^2^

Several clinical and animal experimental studies have shown the significant role played by the function of muscles of mastication in craniofacial growth. It has been shown that relatively large forces are generated when teeth are brought into occlusion and these forces decrease when the bite point is moved anteriorly.^2,3^ There is a controversial relationship between bite force and age and sex of patients. In some investigations no difference between genders was detected, whereas in others males produce greater bite force than the females. Bite force has been shown to increase with age till a specific age and then the levels start decreasing, but the cut-off age for this change is still not known. The variability of the results of bite force has often been considerable with a large number of factors influencing the value obtained.^3,4^

Efforts have been made to investigate the so-called normal chewing forces as well as biting forces in man. Clinical evaluation of the physiologic characteristics of the muscles of mastication by means of accurately measuring the human bite forces have been studied with several types of equipment, and the maximal values reported have varied greatly. Mean values for maximal bite forces in these studies have varied from 216 to 740 N. For the incisal region smaller values, 108 to 293 N, have been reported. Different aspects of bite force have recently been reviewed by Hagberg.^2,4,5^ Various methods, like measurement of myoelectric activity, endurance method and bite force recording, have been used for employing several types of equipment. Extensive studies throughout the world have reported great variance of the recorded maximal values. As need for improvements necessitate, so the continuing endeavor to strive for better, more refined, simpler and accurate device has led to further development in designing a device which can record human bite forces with high level of consistency and accuracy.^6^

The present study describes the design and development of a new novel bite force recorder (gnathodynamometer). In addition, the consistency of this bite force recorder has been reaffirmed by conducting a clinical testing study described in detail in this paper.

## AIMS AND OBJECTIVES

1. To develop a new, more refined, simpler and accurate bite force recorder (gnathodynamometer) for recording relatively large forces, which are generated when teeth are brought into occlusion.
2. To reaffirm the consistency of this bite force recorder by conducting a detailed laboratory calibration test on 30 adult subjects.

## MATERIALS AND METHODS

The bite force recorder consists of a detailed state-of-the-art apparatus which was carefully selected and individually crafted using technical expertise when required.

The actual device was developed in conjunction with the superior technical knowledge as well as advanced armamentarium at Central Scientific Instruments Organization and Precision Tools Galaxy, Chandigarh.

**Fig. 1:**
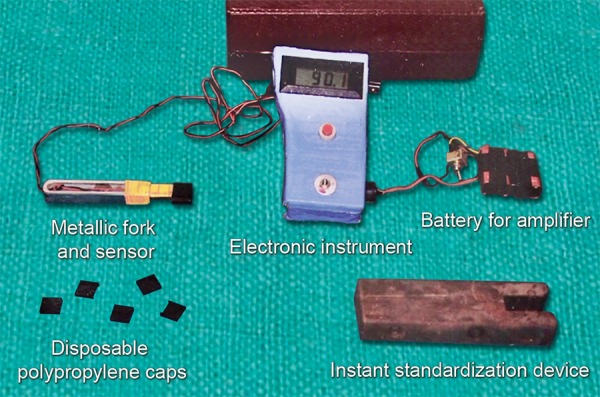
Parts of gnathodynamometer

It consisted of following components (Fig. 1):

- Metallic fork and sensor
- Electronic instrument
- Batteries for instrumentation amplifier, digital panel meter and Wheatstone bridge
- Instant standardization device
- Disposable polypropylene caps.

### Metallic Fork and Sensor

**Fig. 2:**
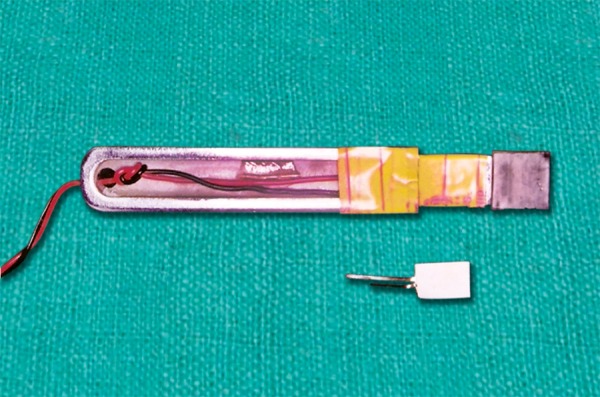
Parts of metallic fork and sensor

A metal housing was constructed in the shape of a fork made with high quality hardened spring steel (Fig. 2). A strain gauge was chosen as a sensory unit which was fixed to one of the surfaces of the fork.

Several dimensions/thicknesses of the fork were tried and the one with optimal springiness and stiffness was finally selected. The total thickness of the fork at the biting end was 9.0 mm and the width was 13.0 mm. There was a gap of 4.0 mm between the upper and the lower parts of fork with each being 2.5 mm thick, thus giving a thickness of 9.0 mm. The total length of the fork was 12.5 cm. The handle was hollowed out to make it light and also for the passage of the wires.

Various milling cum drilling machines and surface grinders were used for making fork and then it was subjected to a normalizing heat treatment ranging from 300 to 450°C for 5 to 6 hours to remove the residual stress incorporated during the entire manufacturing process. This was followed by a hardening and tempering heat treatments at 850°C to 1000°C for 3 to 6 hours to rearrange the carbon content and to increase springiness. Finally, the fork was chrome plated to prevent rusting.

### Electronic Instrument and Batteries

**Fig. 3:**
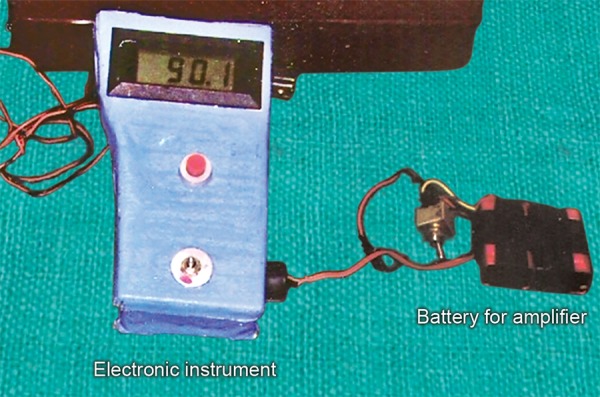
Electronic instrument and batteries

It consisted of a Wheatstone bridge assembly, an instrumentation amplifier and a digital panel meter (DPM) for display (Fig. 3). An adjustable knob was incorporated for bringing voltage to zero (for balancing the Wheatstone bridge) at the start of each recording.

The Wheatstone bridge was supplied by a 1.2 volt battery, the instrumentation amplifier supplied by two ± 9.0 volt batteries and DPM by ± 9.0 volt battery. Whenever a force was applied on fork, it re-enacted a process similar to bending of a beam and caused an elongation of the strain gauge, which caused a measurable change in the resistance of one of the arms comprising the Wheatstone bridge and was ultimately transformed into a change in the recorded voltage which was amplified by an instrumentation amplifier and recorded accurately on the digital panel meter (DPM).

### Instant Standardization Device

**Fig. 4:**
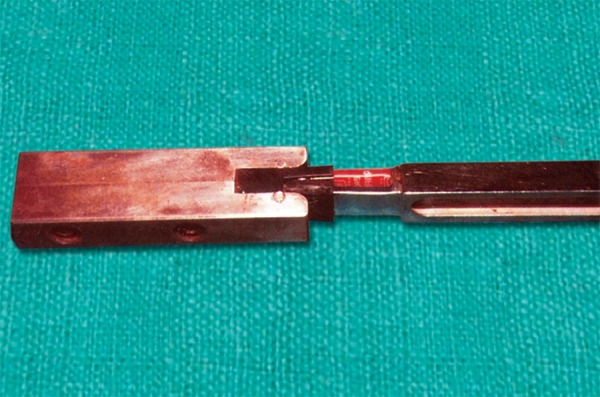
Instant standardization device

The fork was calibrated twice by an instant standardization device just prior to measuring the bite force each and every time (Fig. 4). It consisted of a block of carbon steel with a slot cut at one end, thus possessing high stiffness, minimum deflection and high strength. The slot of the device was smaller than the total thickness of the fork (8.8 mm). The standardization was done at three force levels:

- For light force, when the fork was inserted into the slot without disposable plastic cap, which showed a DPM reading of 2.5 mV
- For medium force, when fork was inserted with one plastic cap and showed 19 to 20 mV on DPM
- For heavy force, which showed 61 to 62 mV on DPM when inserted with both disposable plastic caps.

### Disposable Polypropylene Caps

**Fig. 5:**
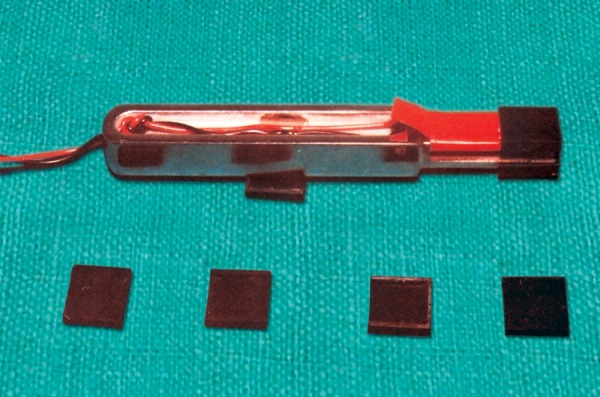
Disposable polypropylene caps

In order to reduce metallic impact on the teeth and to prevent cross contamination, the biting end of the fork was covered with disposable caps, made up of polypropylene possessing good elasticity for ease of placement and removal as well as adequate strength to withstand large biting force without fracture (Fig. 5). The occluding surface of the cap was made slightly convex for good adaptation to the occlusal surface of molars. The thickness of each cap was 1.0 mm, thus increasing the total thickness of the fork to 11.0 mm. There were locks on the inner surface of the cap to snap fit into the notch on fork. For making these caps, a suitable die was made at Precision Tools Galaxy, Chandigarh.

## CALIBRATION TEST

Once the instrument was ready, it was calibrated with loads from 10 to 85 kg by a universal testing machine at Bureau of Indian Standards, Chandigarh. Loads were applied through extracted natural teeth (molars) mounted in acrylic blocks to stimulate the conditions in the mouth. The reading on the DPM was noted for the various force values, and three recordings were taken for each weight. The mean of the three recordings was finally taken.

It was seen that the increase in recorded mV values on DPM were proportional to increase in loads applied.

A regression analysis was applied to find the conversion factor.

Y = a + bX
a = 0 (since curve starts from zero)
b = Constant (gradient/slope of line)
≥ Used for conversion factor
Y= bX = 0.6X

The resultant conversion factor for one DPM recording was taken as 0.6 kg. This was ultimately converted to Newton’s (1 kg = 9.8N ) as 5.88 N.

### Technique

**Fig. 6:**
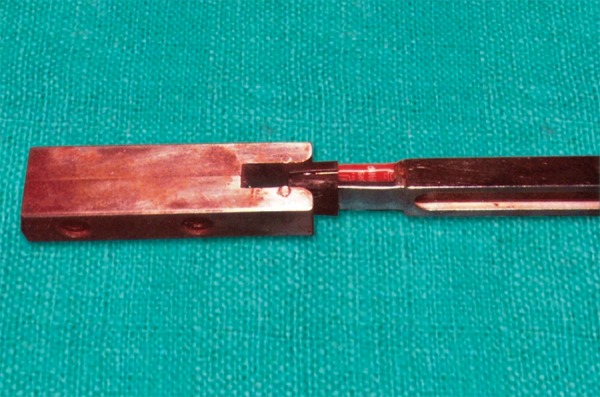
Calibration by instant standardization device before and after each recording

The patients were seated on a dental chair with head unsupported and positioned so that the Frankfort horizontal plane would be parallel to the floor. The patients were explained about the procedure and asked to bite maximally when told.^2,6^ The bite force recorder was calibrated by instant standardization device before and after each recording (Fig. 6).

**Fig. 7:**
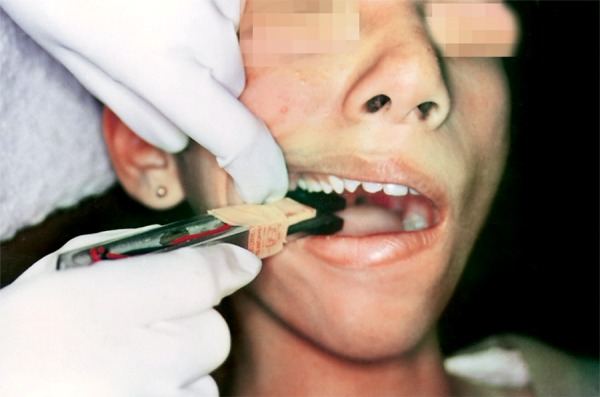
Technique for recording the maximum bite force (MBFP1)

The fork was placed parallel to the dental arch so that biting end was positioned in the right maxillary first molar region (Fig. 7). At the beginning of the test, each subject was asked to bite on the fork in the order to make him familiar with the equipment and no measurements were made.^2^ After that a series of three consecutive recordings were taken and noted. The rest period of one minute was given between each recording to prevent muscle fatigue. Mean of the three recordings was taken as the maximum bite force (MBF) in the molar region (maximal intercuspal position, MBFP1).

**Fig. 8:**
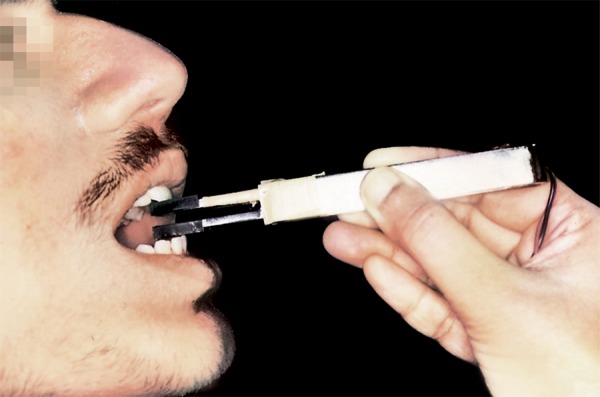
Technique for recording the maximum bite force (MBFP2)

Although in the context of the clinical testing done in this study, the recording of incisal bite position is not required. However, to demonstrate the versatility of design of the bite force recorder enabling multifunction capability and wide array of applications we have described the procedure of recording it also using the same device. In case of recording the incisal bite position^2,6,7^ (Fig. 8), the bite fork was held parallel to the floor and then was carried out to the subject’s mouth, so that the marker on the acrylic pad was positioned against the incisal edges of the maxillary incisors. The subject as instructed to slide the mandible forward, without lateral shift (to establish an end to end incisal edge relationship) and to bite as hard as possible. Mean of the three recordings was taken as the maximum bite force (MBF) in the incisal region (maximal interincisal position, MBFP2).

## RESULTS

### Clinical Testing

- 30 dental students (15 males and 15 females) aged 20 to 25 years volunteered as test subjects in this study to reaffirm the accuracy in recordings of the bite force recorder.
- Criteria for selection
  1. All subjects had full complement of permanent teeth and well-aligned upper and lower dental arches
  2. Stable relationship in maximum intercuspal position
  3. No large and irregular restorations on first molars
  4. No TMJ related disorders.

After detailed study of the sample subjects using the technique described above, the following observations were recorded taking into consideration the statistical variance and calculations (Table 1):

- The mean maximum bite force of the men was 606.8 N (SD = 45.5) with the range of 546 to 668 N
- The mean maximum bite force of the women was 486.2 N (SD = 41.7) with the range of 425 to 546 N.
- Statistically significant difference was observed between genders in the molar/buccal segment/area.

**Table 1:** **Table 1:** Observations recorded of 30 subjects used for clinical testing

*Subjects range*		*Mean MBF*		*SD*	
Males (n = 15) 546-668 N		606.8 N		45.5 N	
Females (n = 15) 425-546 N		486.2 N		41.7 N	

## DISCUSSION

The relationship between form and function of the stomato-gnathic system has been studied by several researchers and it is still not clear, whether a genetically determined facial morphology decides the strength of masticatory muscles or a strong musculature influences the form of the face.

Several clinical and animal experimental studies have shown the significant role played by the masticatory muscle function in craniofacial growth.^2,3,8,9^ To evaluate clinically the physiologic characteristics of the masticatory muscles, various methods, like measurement of myoelectric activity, bite force recording and endurance test, have been used.

Interest in the study of the strength of masticatory musculature dates back to the 17th century as documented by Borelli (1681) who was among the first to measure the force of mastication using a somewhat similar technique of assessing the bite force by transducers placed between one pair of opposing teeth, leaving the rest of the dentition separated.

The most successful of the entire lot of bite force recorders consisted of a metallic fork, an electronic instrument, a instant standardization device and disposable caps. This forms the basis of the framework of the bite force recorder designed entirely in house by us. It has added features, like a strain gauge which was chosen because of its advantages. The total thickness of the fork with disposable caps was 11.0 mm. A similar vertical opening between jaws was produced by recorder used in several researchs. Prior to measuring the bite force, the fork was calibrated by an instant standardization device which is unique to this study. A similar calibration has been reported by Waltimo and Kononen,^2,7^ but it has an inherently different design. To reduce metallic impact on the teeth and to prevent cross contamination, a unique and exclusive set of disposable caps were designed and used by us. The instruments were calibrated with loads from 5.0 to 90 kg by a universal testing machine^2,7,9^. The bite force was recorded by placing the fork in maxillary first molar area as also been done in other studies and was recorded unilaterally.

The human bite forces registered in this study lay within the calibration range. As shown in the results section of this study, the reliability and the validity of the method used in this study are as good as any documented study using state of the art bite force recorder in any part of the medical world.

It was also observed that the measuring range for our bite force recorder was wider than any previously reported for equipment with unilateral housings. This increased range is due to the high-load tolerance capacity of the quartz force transducer used.

There has been controversy surrounding about the differences between men and women as far as maximal bite force is concerned.^2-7,9,10^ In some investigations,^11-15^ no difference between genders was detected, whereas as in others men produced greater bite forces than women. In the present investigation the difference between genders was evident in the maximal bite force recorded in the molar region. This finding is mostly due to the greater muscular potential of men,^16^ because jaw muscle size alone is reported as explaining most of the variation in bite force, although there is recent evidence that similar bite forces can be found in subjects with disparate facial features. Because all subjects were healthy young adults, ranging in age from 20 to 25 years of age, a 2-year difference between mean age of men and women is unlikely to have influenced their maximal bite forces. For men, mean maximal bite force in the molar region might as well represent maximal muscular potential because majority of subjects reported muscle dependent limiting factor.^2,3,5^ For men, it might almost represent their maximal muscular potential because majority of the subjects named a muscle dependent reason, lack of muscular strength or pain in muscles as limiting bite force.

In percentile value, the maximal bite force in the molar region recorded by using our bite force recorder was 41% higher for male subjects than females. This observation can be explained by narrowing down on the fact that men were able to utilize ability for greater bite forces without any notable feedback from the periodontal ligament receptors on the activity of the jaw elevator muscles.

Another interesting observation which was noted was that when the housing was placed along the dental arch, subjects had a tendency as in normal chewing to move the mandible laterally to the side of the housing before his maximal bite force effort. Occasional bite marks on the metal housing indicate that some cusps in heavy biting action penetrated the rubber after which the interocclusal separation fell in the range of favorable clearance of 9 to 20 mm. The rubber caps not only prevent teeth from biting directly on rather uncomfortable and slippery metal surface but also provide sagittal and lateral support to cusps. The stability of the coated housing between teeth may, therefore, partly explain the high bite force values recorded in this study.

## SUMMARY

Bite force could be measured with this new instrument which is completely indigenous. There was also a statistically significant difference between men and women subjects with respect to maximal bite force in the molar region as recorded by the instrument during the clinical authentication trial conducted for testing its accuracy.

Below the comparison of no. of subjects, vertical separation and bite force recording have been given between various studies with that of ours.

It is observed from Table 2 that,

- The results at different studies have been inconsistent and conflicting with wide range and high standard deviations.
- These inconsistent results seen to be lack of control of certain variables^17,18^
  - Variations due to instruments
  - Individual variations.

**Table 2:** **Table 2:** Comparison of different studies

*Author*		*Sample*		*Age group (years)*		*Vertical separation (mm)*		*Bite force*	
Finn Floystrand et al (1982)		8♂-8♀		20-25		4.0		500 N (330-680 N)	
Antii Waltimo et al (1985)		15♂-15♀		20-35		22.0		♂ 847 N (131 N)	
								♀ 597 N (94 N)	
Field et al (1986)		21		24-35		6.0		350N(183N)	
Kiliaridis et al (1993)		37		20-24		11.0		♂ 807 N (140)	
								♀ 650 N (196)	
Bonakdarchian et al (2009)		20♂-20♀		19-27				♂ 73.6 kg (23.8)	
								♀ 53.0 kg (19.6)	
Present study (2009)		15♂-15♀		20-25		10.0		♂ 606 N (45)	
								♀ 486 N (41)	

Variations due to Instruments

- Lack of flexibility of the transducer element
- Dynamic responsiveness and accuracy of the transducer
- Vertical separation of the jaws (size of transducer element)
- Location of the bite force transducer.

Individual Variations

- Jaw morphology
- Degree of physical training or masticatory habits
- Different subject population
- Subjects reluctance to bite maximally
- Head posture, age and sex of the subjects.

## CONCLUSION

To conclude we can exclaim with confidence which is backed by results of our scientific study and assessment of authenticated literature that our bite force recorder is more sensitive, accurate, reproducible, compact, battery operated, hygienic due to disposable covers and has the ability to produce accurate readings in a simplified way which is very helpful and suitable for field studies as well as the clinics.
